# Balanced Energy-Aware and Fault-Tolerant Data Center Scheduling

**DOI:** 10.3390/s22041482

**Published:** 2022-02-14

**Authors:** Muhammad Shaukat, Waleed Alasmary, Eisa Alanazi, Junaid Shuja, Sajjad A. Madani, Ching-Hsien Hsu

**Affiliations:** 1 Department of Computer Science, COMSATS University Islamabad, Abbottabad Campus, Abbottabad 22060, Pakistan; shaukat@cuiatd.edu.pk (M.S.); junaidshuja@cuiatd.edu.pk (J.S.); madani@comsats.edu.pk (S.A.M.); 2 Computer Engineering Department, College of Computer and Information Systems, Umm Al-Qura University, Makkah 21955, Saudi Arabia; wsasmary@uqu.edu.sa; 3 Department of Computer Science, Umm Al-Qura University, Makkah 21955, Saudi Arabia; eaanazi@uqu.edu.sa; 4 Department of Computer Science and Information Engineering, Asia University, Taichung City 41354, Taiwan; 5 Department of Medical Research, China Medical University Hospital, China Medical University, Taichung City 406040, Taiwan; 6 Guangdong-Hong Kong-Macao Joint Laboratory for Intelligent Micro-Nano Optoelectronic Technology, School of Mathematics and Big Data, Foshan University, Foshan 528000, China

**Keywords:** cloud computing, networks, data center, fault tolerance, energy efficiency

## Abstract

Fault tolerance, performance, and throughput have been major areas of research and development since the evolution of large-scale networks. Internet-based applications are rapidly growing, including large-scale computations, search engines, high-definition video streaming, e-commerce, and video on demand. In recent years, energy efficiency and fault tolerance have gained significant importance in data center networks and various studies directed the attention towards green computing. Data centers consume a huge amount of energy and various architectures and techniques have been proposed to improve the energy efficiency of data centers. However, there is a tradeoff between energy efficiency and fault tolerance. The objective of this study is to highlight a better tradeoff between the two extremes: (**a**) high energy efficiency and (**b**) ensuring high availability through fault tolerance and redundancy. The main objective of the proposed Energy-Aware Fault-Tolerant (EAFT) approach is to keep one level of redundancy for fault tolerance while scheduling resources for energy efficiency. The resultant energy-efficient data center network provides availability as well as fault tolerance at reduced operating cost. The main contributions of this article are: (**a**) we propose an Energy-Aware Fault-Tolerant (EAFT) data center network scheduler; (**b**) we compare EAFT with energy efficient resource scheduling techniques to provide analysis of parameters such as, workload distribution, average task per servers, and energy consumption; and (**c**) we highlight effects of energy efficiency techniques on the network performance of the data center.

## 1. Introduction

Data centers are very large computing facilities comprising thousands of servers, hundreds of switches, and Gigabit communication links, storage systems, redundant power supplies, and environment controlling units. Data centers are being extensively used for internal operations and Information Technology (IT) services of government organization, Internet service providers (ISP), commercial IT services providers, cloud computing service providers, industrial units, and educational institutions. Two types of application are common in data centers, (**a**) outward web content, e.g., video streams, web pages, and (**b**) internal processing, e.g., indexing, jobs scheduling [1,2].

A data center should be highly reliable, scalable, redundant, fault-tolerant, and cost-effective. According to a study of outages at U.S.-based data centers by the Ponemon Institute, a failure in a data center may cost up to USD 5600 per minute. Similarly, the downtime can cost an average of USD 505k per incident in data centers [3]. To keep the minimum downtime resources are installed in redundant fashion. These resources consume huge amount of energy, so low-power CPUs, energy efficient supplies, resource virtualization, and smart cooling practices are used to cut power consumption [4,5]. Data center devices tend to fail during early deployment or when becoming obsolete. The cost of failure may increase in the case of enterprise data centers that delivering services to e-commerce and telecommunication sector. Such outages cost more than USD 1M for a single incident. In a data center, different types of failures may occur as detailed in Table 1 [3].

In data centers, high-end computing and redundancy are the two main parameters being considered for high performance and reliability. These two parameters help to tolerate against faults and varying traffic patterns. Energy efficiency techniques applied in data centers are tuning several parameters and applying some power controlling hardware to minimize the wastage of energy. In this scenario, we need to identify the tradeoff between energy efficiency and fault tolerance. There is a need to investigate about the level of redundancy required and the resulting cost of the data center network. Similarly, the tradeoff of energy efficiency schemes and fault tolerance needs to be investigated. Careful consideration about various energy efficiency mechanisms can produce interesting results in terms of the operational cost of data centers without affecting the overall reliability of the data center [6,7].

Data centers consume significant amounts of energy during their operations. Approximately 20–30 TWh/year power demand is being reported by telecommunication sector, networking equipment, and data centers in the USA. This may cost between USD 2.5 to USD 4 billion per year [8]. Data centers and IT equipment contribute 2.4% of total worldwide electricity that is expected to double after every five years at the current rate [9]. Similarly, an exponential traffic increase is being witnessed over networks where more than 60% of traffic is of high-definition video streaming and video on demand. Traffic patterns of data centers are reflecting substantial increase in traffic flows day by day [10].

Energy efficiency is one of the significant topics in large-scale computing systems such as data centers. Techniques such as Adaptive Link rate, Dynamic Power Management, Dynamic Voltage/Frequency Scaling [11], Energy Efficient Ethernet (802.3az) [12], Hardware Assisted Sleep, Low Power Idle, and resource virtualization [8] are being extensively explored in large-scale data centers. Energy efficiency techniques have significant tradeoffs over reliability, performance, and fault tolerance and needs to be evaluated in detail [13,14].

Energy-efficiency-focused resource scheduling techniques may cause the degradation of reliability and performance or may lead to service-level agreement (SLA) violations [15]. On the contrary, more focus on fault tolerance may lead to significant increase in the energy consumption. Once the tradeoff between energy efficiency techniques and fault tolerance is identified, a much better, simpler, and more realistic scheduling technique for data centers can be designed. The focus of this study is to propose a balanced network scheduling technique between the two extremes of energy efficiency and fault tolerance in data center networks. One level of redundancy for fault tolerance with energy-aware computing can be an acceptable solution to have optimum performance, fault tolerance, and energy efficiency in data centers. The proposed energy-aware fault-tolerant scheduling technique can accommodate most of the data center workloads with significant energy saving and failure management. The main contributions of this study are:

- Design of EAFT scheme that reduces energy consumption in data centers while preserving a predefined level of fault tolerance;
- Study the trade-off between energy consumption and fault-tolerant for the balanced EAFT scheme;
- Investigate the energy and data center performance parameters of EAFT with existing energy efficiency techniques.

The rest of the article is organized as follows. In Section 2, we list the related works towards energy efficiency and fault tolerance in data centers. Section 3 describes the main components of proposed EAFT framework along with the algorithms. In Section 4, the details of evaluation methodology and simulation environment are listed. Section 5 details the result of proposed EAFT framework while comparing it with the green (energy-efficient) techniques. Section 6 provides the concluding remarks.

## 2. Related Work

Many researchers suggested scalable and cost-effective network architectures, e.g., Fat-tree, DCell, BCube, and HyperBCube [16]. Data center network architectures are supposed to recover automatically from common failure, e.g., link failure, switch failure, and server failure. Most of the data center topologies deploy redundant hardware to provide fault tolerance and reliable connectivity [17]. The routing protocols provide fault-tolerant data traffic flows on top of redundant infrastructure. The energy efficiency techniques in data centers can be broadly classified into (**a**) Dynamic Voltage and Frequency Scaling (DVFS) (**b**) and Dynamic Power Management (DPM). The DVFS techniques focus on energy conservation ignoring the reliability and fault tolerance requirements of data center networks [18]. Virtualization techniques may also burden the network resources and impact the throughput and latency [19,20].

Liu et al. [21] conducted a detailed comparison of fault tolerance characteristics of different data center topologies. The Fat-tree, BCube, HyperBCube, and DCell architectures are evaluated with metrics including aggregated bottleneck throughput (ABT), average path length (APL), and connection failure rate (CFR). Simulation results show that the Fat-tree and BCube have very high ABT while because of high redundancy. BCube ABT degrades more gracefully than other architectures. In HperBCube and DCell, the switching and wiring cost are lower and have relatively lower ABT than Fat-tree and BCube. In Fat-tree, all backup routes have relatively same length and random failure does not affect path length. The APL of Fat-tree shows no significant change while APL grows significantly with failures because detours need to be taken in other topologies. The Fat-tree topology suffers very much from node and link failures the connection failure rate is recorded relatively high in fat-tree because of low redundancy between switches and servers.

Couto et al. [22] provided a comparative study of reliability and fault tolerance characteristics of major topologies of data center. The study analyzed the tradeoff of data center topologies in context of failures. The switch link and server failures are focused to draw a baseline for the reliability analysis excluding the other factors such as routing algorithms, applications, and traffic engineering strategies. The topology is modeled in the form of an undirected graph. The network nodes and edges were removed randomly to analyze the effects of failure. The study focused on performance degradation in a situation where faulty devices are not repaired. Different data center topologies were simulated with the number of servers as high as 2400 to represent a typical data center. The hybrid topologies such as BCube and DCell have smother decays than Fat-Tree. After certain failure ratios, hybrid topologies tend to disconnect more frequently, while the fat-trees tend to be more reliable in higher failure rates. On the other side, the server in fat-tree topology tends to disconnect in the case of immediate link failure, while in hybrid topology such as DCell, servers have better connectivity having multiple connection with multiple ports. In Fat-tree, switch failure shows more disconnectivity than other hybrid topologies. The server failures affects more on hybrid topologies having reliance on servers to forward packets to other subset of network.

Phillipa Gill et al. [23] conducted a very detailed analysis of failures in a data center networks. The focus of the study was to identify the most failure-prone devices or links, the causes of failures, identifying the failures having impact on network traffic, and the effectiveness of network redundancy. SNMP alerts, syslog messages, error logs, and trouble tickets of one year were collected from geographically distributed data centers sources to study the behavior of failures. Three-tier data center networks having 1:1 redundancy at the core and distribution layers was focused as a base topology, having redundant load-balancer at distribution layer. Data center workloads are identified as large volumes of mice flows (short-lived latency-sensitive) and a few elephant flows. The study concluded that data center networks and commodity devices show high reliability with most of the failures occurring on the load-balancers. Moreover, the redundant network devices can only lower the median impact of failure by 40%.

Most of the research in data center resource scheduling either focuses on energy efficiency or reliability, while ignoring the tradeoff of the other. Energy efficiency techniques proposed under the dynamic power management (DPM) totally ignore the impact of network or server failures [24,25]. The ElasticTree [26] proposed an efficient energy conservation technique that only addresses the fault tolerance with respect to the controller and optimizer modules. The GreenCloud [27] and DENS [28] scheduler proposed workload consolidation based on energy efficiency techniques (DNS and DVFS). The aforementioned resource schedulers also ignored the impact of failure and fault tolerance requirement in data center networks. As per best of our knowledge, no research work so far has highlighted the tradeoff of energy efficiency and fault tolerance in data center networks.

## 3. Energy-Aware Fault-Tolerant Data Center Scheduler (EAFT)

The EAFT methodology incorporates the requirements of energy efficiency and fault tolerance in data center networks. It minimizes the overall impact or tradeoff of one data center performance metric between the two extremes. It presents a realistic and practical way of achieving the best of both energy efficiency and fault tolerance. EAFT task scheduling achieves a fair amount of energy efficiency without violating the other factors such as SLA, throughput, delay, and performance. Similarly, the resource management of the EAFT provides one-level fault tolerance for better user satisfaction and availability requirement of data center network. An arbitrary level of redundancy can be utilized in the evaluation of the EAFT scheduler. However, recent research studies indicate that one level of redundancy is enough for balanced performance in most cloud data centers [29,30]. The requirement of data center fault tolerance can vary in the nature of applications. Therefore, our modular EAFT approach can be extended for application-specific analysis. The subsections below will detail the EAFT algorithm, failure generation model, and EAFT data center network architecture.

### 3.1. EAFT Algorithm

The flow of operations in EAFT scheduler is different from Green [27] and DENS methodologies [28]. Green and DENS schedulers focus only on energy efficiency. The EAFT scheduler provides energy-aware, one-level fault tolerance scheduling in data centers. To measure the performance of the EAFT scheduler, we also incorporate realistic random faults at various levels of a data center to examine more realistic scenarios. The self-explanatory pseudocode for the EAFT scheduler is provided in Algorithm 1.
**Algorithm 1** Pseudo code for EAFT.1:Input: Task_Dist_Percentage, Failure_Rate, Simulation_Time (T), Data center topology2:Output: EnergyAware Fault Tolerant topology3:WORKLOAD SCHEDULING: As per Task_Dist_Percentage4:APPLY: Node failure generation algorithm5:MAINTAIN BACKUP: At each layer of data center network6:CALCULATE: New Topology7:APPLY: Energy Efficiency Technique(s) to idle resources8:**repeat**9:   FORWARD: Traffic flows10:   **if** FAILURE OCCURS **then**11:     NOTIFY: The topology change12:     RECALCULATE: The shortest path and shift the traffic13:     ACTIVATE: New backup path (if required)14:   **end if**15:   **if** FAILURE RECOVERS **then**16:     NOTIFY: The topology change17:     RECALCULATE: The shortest path and shift the traffic18:     APPLY: Energy Efficiency Technique(s) (if required)19:   **end if**20:**until** T

The Task_Dist_Percentage reflects the average workload distribution of a data center per server. The Failure_Rate is the fault-tolerance level required for a data center. The EAFT employs one fault-tolerant (redundancy) level to provide both energy efficiency and reliability. The EAFT algorithm takes an average task distribution of the data center, data center topology, device failure rate, and simulation time as input and calculates a balanced energy-fault-tolerant topology. The failure generation algorithm marks random devices for failure. The network flows are adjusted after each node failure according to backup redundant paths. The equal cost multi-path routing protocols running in default data center scenarios help in the redistribution of flows in the case of network node failures [31]. The network topology changes and the shortest path calculations are made accordingly.

### 3.2. EAFT Failures Model

We developed a TCL (Tool Command Language) script based on the Failure_Rate to introduce device-level failures at each layer of the data center topology in the EAFT model. Our failure model is topology-aware; it first checks the type of topology we are using and then introduces random failures at each layer by calculating the number of failures based on the failure threshold value and the total number of devices at that layer.

For example, if we have X number of switches at distribution layer and the failures threshold is A%, it will generate a random number less than or equal to the A% of X, so it may have Y number of devices failing with such circumstances. After that, the Y number of devices will be randomly selected to simulate the effects of failure, each device will fail at time K for L seconds that must be less than the total simulation time. For random number generation, we utilized uniform distribution to simulate equal chances of failure for all devices. The same process is repeated for all data center layers to generate failures. It is not compulsory that failure will occur every time as we may obtain the value 0 from the rand() function. Such a condition will project the no failure scenario. The pseudocode for failure generation is provided in Algorithm 2:
**Algorithm 2** Pseudocode for Node Failure Generation.1:INPUT: T(start), T(end), Failure_Rate, Data center topology2:OUTPUT: Random device failures at each layer3:**for** Each layer in Data Center Network **do**4:   SET Num(F) := (A random number <Total No of nodes at layer * Failure_Rate)5:   **if** Num(F) >0 **then**6:     **while** Num(F) <0 **do**7:        F_Node := (A random number < Total No. of nodes at layer)8:        F_ST := (A random start time <T(start) )9:        F_RT := (A random recovery time <T(end) )10:        NodeFailure (F_Node failing at time F_ST till F_RT)11:        Num(F) := Num(F) - 112:     **end while**13:   **else**14:     DISPLAY “No Failure at Layer”15:   **end if**16:**end for**

Where Num(F) is the number of failures. All other variables are described in the above-mentioned pseudo code. The failure node generation procedure takes the simulation time, the data center topology, and the failure rate as inputs. For each layer of the data center topology (access, distribution, and core), the devices are randomly selected for failure based on the given failure rate. As the failure rate remains constant for a simulation instance, the failing nodes remain in the same state for the duration of simulation. For varying cases of failure rates, the simulations are repeated with different values of Failure_rate.

### 3.3. EAFT Architecture

**Figure 1:**
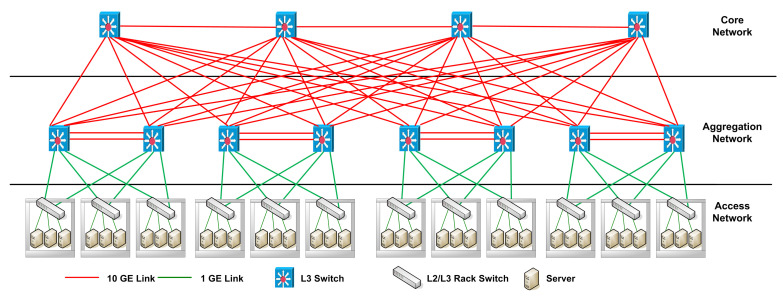
EAFT Architecture with one level of fault tolerance.

The structure of the EAFT in Figure 1 is designed over traditional three-tier data center topology illustrated in figure [32].

EAFT architecture inherits all major strengths and characteristics of the GreenCloud scheduler. On top of the Green scheduler, the Task Scheduler module is modified to introduce a more realistic workload consolidation. The task scheduler schedules the workload equally to a set of severs. The computation resources such as servers are utilized in a more realistic manner having an average CPU load of 75%. The scheduler avoids any possibility of CPU overheating, CPU overloading, and keeps a room for some short-lived time-critical workloads or for some internal processing of data center network. Mice or elephant work-flows that arrive after task scheduling algorithm execution can be scheduled over underutilized or idle computation resources available without any delay. This type of task scheduling also distributes the traffic flows to switching fabric in a more realistic manner. Taking full advantage of the ECMP routing, the congestion reported by DENS is mostly avoided. The task scheduler schedules the tasks on servers and defined the paths of flows in such a highly practical manner that it reserves a fair set of switches and servers to manage with device or link failures.

## 4. Evaluation Methodology

The Data Center Network (DCN) topology utilized in the evaluation is listed in Table 2. The DCN topology details the number of servers, switches, and users utilized in all simulations.

We have evaluated the power consumption servers and switches in three-tier topology arrangements, which is the most common and accepted topology for data centers. Rack servers, core switches, aggregate switches, and access switches are the main IT infrastructures in three-tier data centers. Over large periods of time, studies have found the average data center workload to be at 30% [27]. Therefore, in all our simulation scenarios, we utilized the average data center load of 30%. GreenCloud and DENS, which have been utilized in the comparison, employed similar simulation parameters without incorporating the failure scenarios. GreenCloud focuses on energy efficiency while ignoring other performance parameter. On the other hand, the DENS schedules the servers at a lower utilization level than GreenCloud to accommodate network performance. We evaluated our EAFT scheme in the GreenCloud simulator. GreenCloud is built over the NS3 network simulator to capture the energy consumption of conventional tiered data center networks. The GreenCloud simulator captures packet-level details with a GUI summarizing the results of simulations. The energy consumption in GreenCloud simulator is calculated based on simplified energy consumption models of servers and switches and the total simulation time.

**Figure 2:**
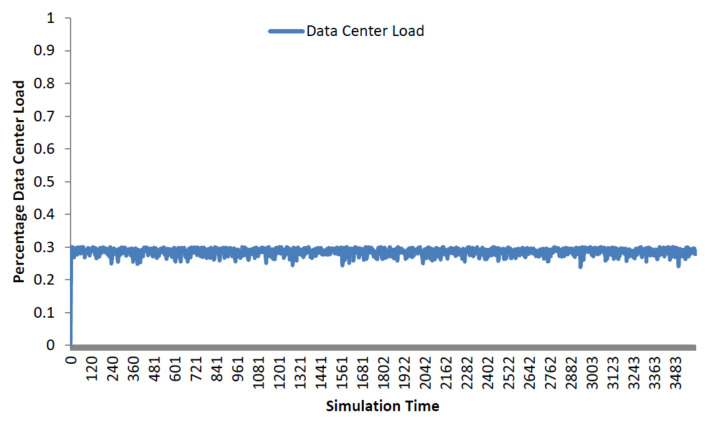
Average Data Center Workload.

The power model of the server components is dependent on the server state and its CPU utilization. An idle server consumes about 66% of the energy compared to a fully utilized server. The DVFS enables a tradeoff between the computing performance and the energy consumed by the server. The DVFS is based on the fact that switching power in a chip decreases proportionally, as defined in Equation (1):

(1) $$P=P_{f}+V^{3}.P_{fixed}$$

where $P_{fixed}$ is the portion of the power consumption that does not scale with the operating frequency *f*, while $P_{f}$ is a frequency-dependent CPU power consumption. The power efficiency of DVS-enabled links is controlled as only 3–15% of the consumed power scales linearly with the link rate. The energy consumed by a switch and all its transceivers is defined in (2):

(2) $$P_{switch}=P_{chassis}+n_{linecards}+p_{linecard}+\sum_{i=0}^{R}n_{ports,r}+P_{r}$$

where $P_{chassis}$ is related to the power consumed by the switch hardware, $P_{linecard}$ is the power consumed by any active network line card, and $P_{r}$ corresponds to the power consumed by a port running at the rate *r*. Further details of energy models can be found in the GreenCloud study [27]. Figure 2 shows the data center load of our simulation scenarios.

## 5. Results

In this section, we discuss the results of EAFT while comparing it with GreenCloud [27]. We chose GreenCloud for comparison as (**a**) we utilized the same simulator as GreenCloud, and (**b**) GreenCloud is one of the most cited energy efficient resource scheduling technique. The results include the comparison of workload distribution, energy, and failure impacts in different scenarios.

### 5.1. Workload Distribution

**Figure 3:**
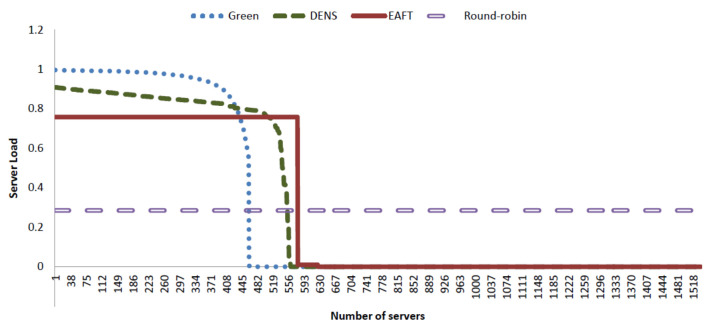
Data Center Workload Distribution.

Workload distribution at servers is the main element that describes how computation work will be performed by servers in data center, it depicts the usage pattern of the servers according to the load. Figure 3 presents the workload consolidation pattern of our EAFT approach along with the comparison with other existing schemes.

All schedulers are considering the average workload from 28.5% to 30%. Round robin is the most reliable scheme fulfilling the fault tolerance requirement for any data center. It is also widely accepted for its full potential for load balancing, 28.2% of the data center load is successfully distributed over all 1536 servers of the data center. There is no problem of overloading, overheating, and failures because the failures can easily be accommodated in this scenario. If a rack of servers or network devices fail, other servers have plenty of resources to accommodate the load without causing any delay. However, the round-robin scheme has several disadvantages, such as the under-utilization of resources and high electricity bills. Round-robin is highly reliable and fault-tolerant but extremely inefficient as for as the energy consumption is concerned.

The Green scheduler exponentially distributes the work-flows among a set of servers, employs DNS and DVFS schemes to conserve energy. It overloads the average load of 30% to approximately one-third of the total servers exponentially and applies DVFS on the less-utilized servers. Hence, the Green scheduler saves two-thirds of the server’s energy resources. The Green scheduler is the most energy-efficient scheme as it ignores all other parameters of data center performance such as fault tolerance. The Green scheduler is not practical for the real data center as it does not consider performance requirements, such as, reliability, fault tolerance, and SLA violations.

To overcome the problems of the Green scheduler, a DENS scheduler was proposed. The DENS scheduler starts distributing workloads by overloading only 90% of the server CPU and 95% of the switching fabric. Hence, the DENS utilizes more servers for computation workloads than Green scheduler. The main focus of the DENS was over-subscription ratios and queue congestion over switches. The DENS significantly solves the problem of network congestion at the cost of a slight increase in energy consumption. The DENS also suffers from other disadvantages of the Green scheduler, having very little consideration over the reliability of the system and fault tolerance. Overloading of 90% of the CPU is still not a good idea for servers running 24/7. Like the Green scheduler, the DENS scheduler also does not comply with the industry standards of SLA requirements.

EAFT incorporates a more realistic subset of computation and switching resources to provide the industry compliance data center performance with reliability, fault tolerance, and SLA requirements. The computation resources are only 75% overloaded to accommodate 30% of the average incoming workload while the DNS is applied on remaining idle resources for overall energy conservation. EAFT scheduler can be seen as a hybrid of DENS and round-robin that receives major benefits of both techniques. EAFT does not overload the servers and keeps 25% of the room for internal processing to accommodate time-sensitive, short-lived mice flows entering at random times.

EAFT leaves one rack of servers powered on to accommodated complete server or rack failures. The EAFT scheduler maintains workload distribution using a threshold (e.g., 75% workload) value. Each incoming flow can easily be adjusted to an underutilized server without any complex calculation. Such scheduling also maintains a fair use of CPU all the time and increases the life span of all servers equally.

### 5.2. Energy Efficiency

In this subsection, we present the detailed analysis and results of power consumption and energy-efficiency-related aspects of Green, DENS, Round-robin, and EAFT scheduling techniques. Table 3 shows the breakdown of power consumption of switches at different layers and servers along with the overall comparison.

Network devices and computation servers are two major power-hungry resources in the data center. In three-tier data center network topology, core, aggregation, and access switches are arranged in highly redundant manner. The electricity consumption listed in Table 3 details network switches at each layer and rack servers.

The round-robin scheduler is most expensive in terms of power consumption at each layer. The EAFT scheduler in comparison with Green and DENS schedulers is more costly at all layers because of the introduction of one-level fault tolerance. Power consumption at access layer is relatively equal among the Green, DENS, and EAFT schedulers. However, the round-robin technique is again more expensive than other techniques at the access layer. At the network layer, the Green, DENS, and the EAFT schedulers consume 33%, 34%, and 42% less energy compared the round robin scheduler, respectively.

In comparison to power consumption of switching equipment, servers consume much more electricity. We found that the power consumption among these two types of IT equipment is 1:8 to 1:11 approximately. Among the IT equipment, the servers consume most of the power in the data center. Our results show that the round-robin technique that applies redundant resources consumes 409.758 kWh of energy. The Green scheduler is most energy efficient and saves about 63% of power as compared to the round robin scheduler. DENS incorporates QoS requirements to fight against congestion-related losses and preserve about 59% of power resources. The EAFT approach consolidates the workload in highly balanced manner, taking significant consideration towards redundancy, fault tolerance, and SLA requirements of data center while saving reasonable electricity. EAFT saves about 55% of the data center’s energy as compared to the round robin scheduler.

### 5.3. Impact of Random Device Failure

The device failure may or may not have impact on the performance parameters of the data center. According to recent studies, the average start-up time of a Linux VM is about 44.2–96.9 s, and the average start-up time of a Windows VM is 429.2 to 810.2 s. For communication fabric, the switches may take 30–180 s to return to the ON state from the OFF state, which may increase latency and suffers from significant loss of data flows [33]. Therefore, DNS scheme may affect the throughput and end-to-end delay in case there is no backup path available to reach destination.

#### 5.3.1. Number of Backups

**Figure 4:**
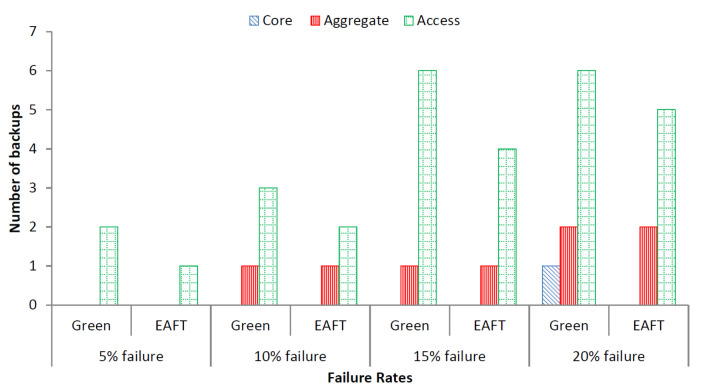
Three-tier random device failure pattern.

We simulated random number of device failures that is less than the failure threshold value at each layer (pseudocode is given in Section 3). After obtaining the random value for the number of failures, we again choose random devices to fail at random time during the simulation. The same scenario is simulated for both the Green scheduler and the EAFT schemes. We exclude DENS and round robin schedulers from further analysis. The round robin scheduler provides the basic comparison for energy and workload distribution. While the DENS scheduler is an extension of the Green scheduler and can be ignored in further analysis. The average number of network devices required to compensate a percentage of failures as per the random distribution are depicted in Figure 4.

We observed that most the failures occur at access layer because of having more quantity of switches as compared to core and aggregate layer. Moreover, the Green scheduler requires more network devices for backup as the existing devices work at almost 100% utilization. The EAFT scheduler requires the least number of backup devices in case of failure as existing devices are running at approximately 75% load, hence, can handle more load without requiring backup devices. The result shows that the EAFT scheduler requires a 27% lower number of network switches in comparison to the Green scheduler to compensate for device failures.

#### 5.3.2. Data Center Load

**Figure 5:**
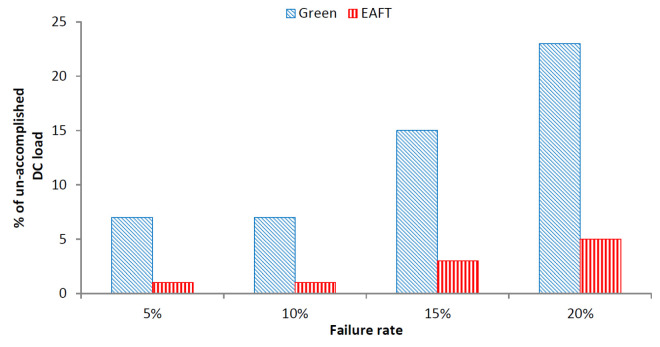
Failure Impact on DC Load.

The impact of failure on the data center’s overall load is one of the unavoidable matter for the justification of EAFT scheme. Figure 5 depicts the failure impact corresponding to the number of the unfinished task.

The result shows that for increasing failure rates, the percentage of unfinished tasks increases. Therefore, there is also a decrease in the average data center workload accomplished. Moreover, the percentage of unfinished tasks is less for the EAFT than the Green scheduler in all failure cases. As the EAFT employs more data center resources at lower utilization levels than the Green scheduler, it can handle failures with respect to the data center workload more efficiently. Overall, the Green scheduler has 80% more unaccomplished tasks as compared to the EAFT in all of the failure scenarios.

#### 5.3.3. Average Task per Server

**Figure 6:**
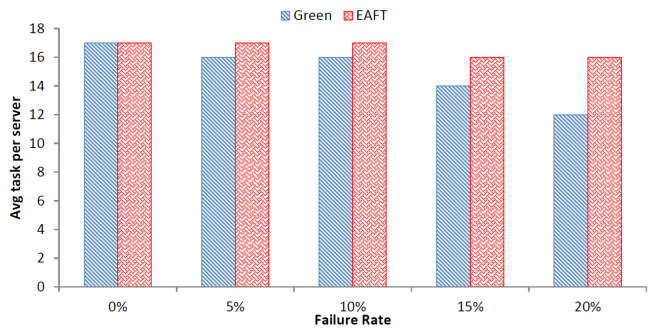
Impact of Failure on Average Tasks per Server.

The increase in the failure rate can lead to the decrease in average tasks per server of the data center. This can be incurred from the Figure 6 depicting the impact of failure on average tasks per server.

As the failure rate increases, the average task per server for the Green scheduler decreases rapidly. On the contrary, the average task per server for the EAFT remains stable. The average task per server is 75% less for the Green scheduler than the EAFT in the worst case. The average task failure per server for the EAFT is 1.1% as compared to 6.5% for the Green scheduler over the increasing failure rate. The EAFT scheduler provides stable task per server rate in case of failures as compared to the Green scheduler. Therefore, the EAFT scheduler will lead to lesser number of SLA violations in case of failures in a cloud data center.

#### 5.3.4. Energy Consumption

**Figure 7:**
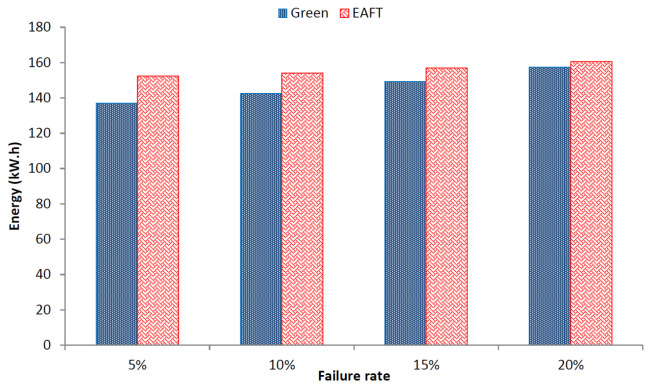
Impact of Failure on Energy Consumption.

In previous subsections, we provided an overall view of energy consumption for the schedulers considered in comparison. In this subsection, we provide the energy consumption in failure scenarios. The energy consumed by the data center topology tends to decrease with the increasing failure rate. Figure 7 provides a comparison of EAFT and Green scheduler energy consumption with increasing failure rate.

The aforementioned result shows that the energy consumption decreases as the increasing number of devices fail. However, the decrease in energy of the Green scheduler is higher than the EAFT. This is due to the fact that the number of devices failing in the case of the Green scheduler are more than the EAFT as detailed in the previous subsection listing the impacts of failure on the number of backups. The energy consumption of the EAFT remains relatively stable over the course of increasing device failure rate. On average, the Green scheduler consumes 13% more energy than the EAFT over the increasing failure rate scenarios.

#### 5.3.5. Impact of Failures on Average Throughput

**Figure 8:**
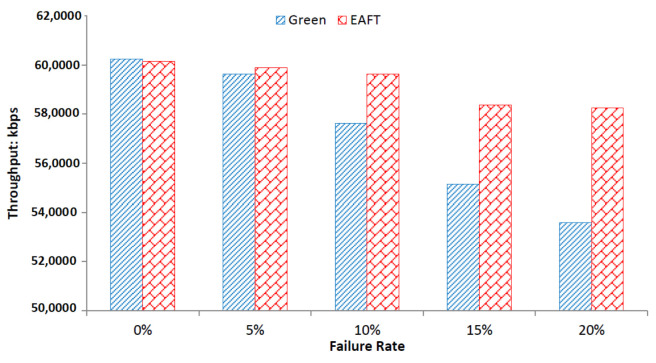
Impact of Failure on Average Throughput.

Failure rates can effect the network performance of a data center aggressively. The average throughput is one of the important network parameters to justify the applicability of any resource scheduling technique in cloud data centers. Figure 8 compares the average (non-instantaneous) throughput of the data center for Green and EAFT scheduler.

The throughput of the EAFT scheduler is very slightly affected by the increasing failure rate from 0% to 25%. The average throughput decrease on increasing failure rate is 0.78% for the EAFT. On the other hand, the Green scheduler’s throughput fluctuates highly with the increasing failure rate. The average throughput decrease rate for the Green scheduler is 2.2% over the increasing failure rate. The EAFT scheduler shows stability in the network performance over the Green scheduler in this case.

## 6. Conclusions

The average workload of the data center remains at 30%, motivating the need to develop energy-efficiency techniques. It has been observed that energy efficiency techniques do not consider fault tolerance. Therefore, we proposed an Energy-Aware Fault-Tolerant (EAFT) technique in this work. The EAFT employs a balanced workload consolidation technique that neither suffers from energy inefficiencies nor from fault-related problems. We incorporated a uniform workload consolidation technique to maintain a workload of approximately 75% at each server to accommodate the average workload of 30% of overall data center. This approach slightly increases the number of active servers and utilizes more energy than the Green scheduler. However, the EAFT scheme does not suffers from overloading, SLA violation, and network convergence problems. For fault tolerance, the EAFT reserves enough subsets of communication and computational devices. We implemented a fault model to introduce random failures at each layer of the data center network. We evaluated the results of 0% to 20% failure rates with Green and EAFT schedulers. The EAFT scheme shows better network performance in the form of higher average throughput. The average throughput decrease rate for the Green scheduler is three times higher than that of the EAFT. The average tasks per server decrease at a six times higher rate for the Green scheduler as compared to the EAFT. Similarly, the Green scheduler has 80% more unfinished tasks as compared to the EAFT over the increasing failure rate. For future work, two research directions are of high importance. First, we used one-level redundancy in this work. The EAFT framework needs to be enhanced to investigate the trade-off between energy consumption and fault tolerance for multi-level redundant data center networks. Secondly, the GreenCloud Simulator provides a three-tier data center architecture. BCube and DCell architectures need to be investigated for similar fault and energy balanced resource scheduling methodologies.

## Figures and Tables

**Table 1:** Causes of Unplanned Outages in Data Centers.

Unplanned Outages	% of Failures
Weather problems/issues	12%
UPS battery failure	29%
Power generator failure	10%
Human/configuration error	24%
Heat, water, or cooling failure	15%
IT equipment failure	15%
Other issues	5%

**Table 2:** DCN Topology Parameters.

Element	Number
Core Switches	4
Aggregate Switches	8
Access Switches	32
Servers	1536
Cloud users	2

**Table 3:** Data Center Power Consumption in kW.h.

Element	Green Scheduler	DENS Scheduler	EAFT Scheduler	Round Robin Scheduler
Core Switches	5.97	5.97	5.97	11.94
Aggregate Switches	5.97	5.97	8.96	23.89
Access Switches	1.67	1.83	2.16	5.33
Network Switches	13.6 (33%)	13.8 (34%)	17.1 (42%)	41.2
Servers	136.51 (37%)	155.2 (42%)	169.3 (46%)	368.6
Data Center	150.1 (37%)	168.9 (41%)	186.4 (45%)	409.8

## Data Availability

Not applicable.
